# Is coracohumeral distance associated with pain-function, and shoulder range of movement, in chronic anterior shoulder pain?

**DOI:** 10.1186/s12891-017-1498-0

**Published:** 2017-04-04

**Authors:** S. Navarro-Ledesma, F. Struyf, M. T. Labajos-Manzanares, M. Fernandez-Sanchez, A. Luque-Suarez

**Affiliations:** 1 Department of Physiotherapy, University of Malaga, Malaga, Spain; 2 Departament of Rehabilitation Sciences and Physiotherapy, University of Antwerp, Antwerpen, Belgium; 3 Department of Nursing, Physiotherapy and Medicine, University of Almeria, Almeria, Spain

**Keywords:** Shoulder pain, Ultrasonography, Diagnosis, Rehabilitation, Chronic pain

## Abstract

**Background:**

The aim of this study was twofold: (i) to assess the intrarater reliability of coracohumeral distance; (ii) to investigate the level of association between coracohumeral distance measured by ultrasonography, and pain-disability and shoulder range of movement, in patients suffering from chronic anterior shoulder pain.

**Methods:**

An observational, cross sectional study was carried out. A convenience sample comprised of 87 patients with chronic anterior shoulder pain was assessed from 3 primary care centres. Main outcomes as pain and function were measured through the shoulder pain and disability index. Furthermore, shoulder range of movement-free of pain in shoulder elevation, as well as coracohumeral distance at both 0 and 60 degrees, were collected.

**Results:**

Absence of any correlation was found between coracohumeral distance and shoulder pain and disability index at both 0 and 60 degrees of shoulder elevation. Furthermore, absence of any correlation was found between coracohumeral distance measurements and active shoulder range of movement -free of pain.

**Conclusions:**

There was poor association between coracohumeral distance and shoulder pain and function, as well as with shoulder range of movement, in patients with chronic anterior shoulder pain. Hence, clinicians should consider, not only increasing this space, but also other possibilities in their therapies, when patients with anterior shoulder pain are treated.

**Trial registration:**

ACTRN12614000144617. Registered: 1^st^ March 2014.

## Background

Shoulder pain is one of the most common musculoskeletal conditions in primary care, with a prevalence fluctuating from 6.9 to 26% for point prevalence, 18.6–31% for 1-month prevalence, 4.7–46.7% for 1-year prevalence and 6.7–66.7% for lifetime prevalence [1] and with 12-month recurrence rates approximately twice the prevalence rates [2]. In the working population, shoulder pain prevalence associated with musculoskeletal disorders is even higher [3].

Anterior shoulder pain has traditionally been underestimated in the assessment of shoulder pain [4]. Although it can occur alone, it usually presents with anterolateral shoulder pain (labeled as subacromial pain syndrome), sharing similar symptoms [5] and making it difficult to diagnose. The most related cause of anterior shoulder pain is subcoracoid impingement syndrome, defined as the encroachment of the posterolateral coracoid process upon the lesser tuberosity of the humerus [6], causing a compression of soft tissues, such as the subscapularis tendon, glenohumeral joint capsule and subcoracoid bursa, and occasionally the long head of the biceps tendon [7]. Anatomic differences for humerus lesser tuberosity and coracoid process [6, 8], as well as anteversion and internal humeral rotation [7], and a history of chronic overuse of persisted flexion, adduction and internal rotation shoulder positions [9], have also been established as possible causes of anterior shoulder pain.

Diagnosis of anterior shoulder pain has not been widely investigated, but physical examination (cross-arm adduction test) and radiographic features are the most commonly used methods [9]. The coracohumeral interval (CHI) has been measured in previous investigations using the coracohumeral distance (CHD) to determine the severity of anterior shoulder pain [5, 6, 10], sometimes by means of computed tomography or magnetic resonance imaging (MRI). However, there is a clear lack of standard procedure to quantify it.

Ultrasonography (US) is a non-invasive tool without ionizing effects that permits dynamic evaluation, and is more accessible than those previously described. It has been widely used in the determination of the acromiohumeral distance (AHD) [11, 12]. Two studies have investigated the use of US in the evaluation of CHD [13, 14]. Oh et al. recently found a good correlation (>0.7) between US and MRI in quantifying CHD, as well as an excellent intra-rater reliability (>0.90) in patients with rotator cuff tear, supporting the use of US in the evaluation of coracohumeral interval. However, there is a lack of a clear measuring process, normative values and reliability data for CHD, measured by US, in patients suffering from anterior shoulder pain. There is also inconclusive evidence on the association of anterior shoulder pain with pain-function and shoulder range of movement (ROM), in patients with chronic anterior shoulder pain. The role of acromiohumeral distance (AHD) as an explanatory factor for symptoms in RC tendinopathy is starting to be questioned [15]. However, the research about whether CHD could play an important role in the explanation of anterior shoulder pain, is unfinished. If a strong relationship between a reduced CHD and high levels of pain existed, decreased shoulder function and limited shoulder ROM would be identified, allowing preventive and therapeutic efforts to be focused on increasing this space. Hence, the aim of this study was twofold: i) to assess the intrarater reliability of CHD at 0 and 60 degrees of scapular elevation measured by US, in patients suffering from chronic anterior shoulder pain; ii) to determine the association between CHD with shoulder pain, function and shoulder-ROM free of pain.

## Method

### Procedure

A convenience sample of 102 patients with unilateral chronic anterior shoulder pain (more than three months), and with clinical symptoms of anterior shoulder pain, was recruited from three different primary care centers. General practitioners (GPs) carried out the recruitment. Then, research assistants assessed participants for eligibility. If participants satisfied the inclusion criteria, then they were studied. Five participants declined to participate, and 10 participants did not meet the inclusion criteria, hence, a sample comprised of 87 participants was assessed. Research assistants collected the informed consent for every participant.

All participants in the study gave their written informed consent. Participants had to meet the following inclusion criteria to be classified as anterior shoulder pain [9, 16, 17]: i) positive cross-arm test; ii) painful arc of movement during forward flexion and/or internal rotation; (iii) elicitation of tenderness throughout palpation of the coracoid process.

Furthermore, other inclusion criteria had to be met: both men and women aged between 18 and 55 years; no history of significant shoulder trauma, such as fracture or clinically/ultrasonographic-suspected full thickness rotator-cuff tear. Participants were excluded from this study if any of these conditions were presented: (i) recent shoulder dislocation, systemic illnesses such as rheumatoid arthritis, and evidence of adhesive capsulitis, as indicated by passive range of motion loss > 25% in 2 planes of shoulder motion, and loss > 50% in passive external rotation; (ii) shoulder pain that was deemed to be originating from any passive and/or neck movement or if there was a neurological impairment, osteoporosis, haemophilia and/or malignancies; (iii) shoulder surgery in the last year, (iv) corticoid injections during the 6 months prior to the study; (v) analgesic-antiinflamatory medication intake during 48 h prior to the study.

### Outcome measures

#### Coracohumeral distance (CHD)

A diagnostic ultrasound unit, Sonosite M-turbo (GE Healthcare, Wauwatosa, WI) with a dynamic range up to 165 dB, was used. Furthermore, a 6–13-MHz linear transducer with 196 piezoelectric crystals with a specific ultrasound system called “SonoMB® multi-beam imaging”, to increase resolution and improve visualisation of physiological and subtle tissue differences, was used to capture images in a grey scale of 256 shades. Ultrasound images were obtained by a single examiner, who was a licensed physiotherapist with advanced training in musculoskeletal ultrasound imaging, and 4-years of experience. Three measurements were taken. An interval of 1 minute was provided between measures, encouraging the patient to move freely. Patients were then repositioned and the second and third set of measurements was successively taken. The ultrasound examiner was blind to all measurements (values were obscured by placing a sticker on the ultrasound screen, meanwhile a research assistant took them and put into a dataset). All the ultrasound measures were expressed in centimeters. CHD was measured at 0 and 60 degrees of active shoulder elevation in the scapular plane, neutral shoulder rotation, with the participant seated in an upright position.

**Fig. 1:**
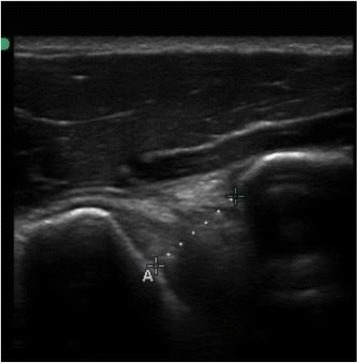
CHD at 0 degrees of shoulder elevation

**Fig. 2:**
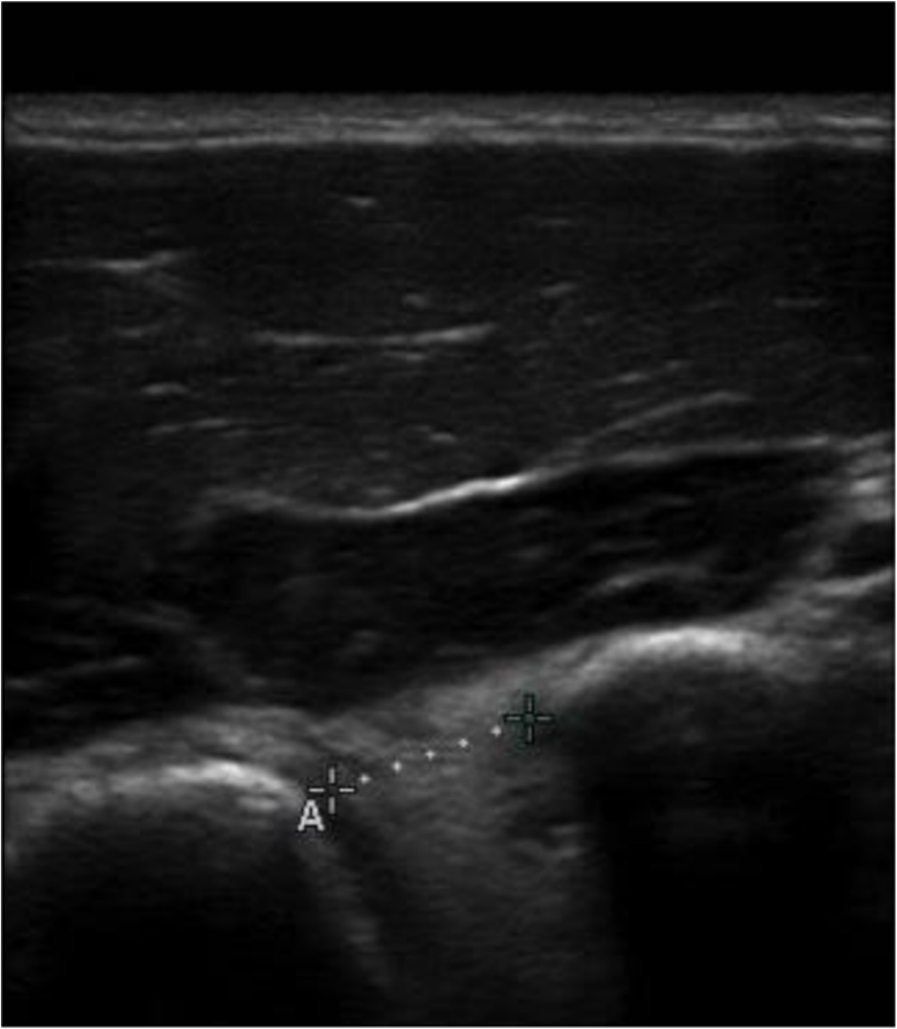
CHD at 60 degrees of shoulder elevation

Patients were seated upright without back support, their feet flat on the ground. To guarantee 0 and 60 degrees shoulder elevation, a hydro-goniometer was placed on the patient’s arm [18]. CHD was defined as the shortest linear distance between the coracoid and the adjacent humeral head [9]. The ultrasound transducer was placed over the most anterior aspect of the shoulder, observing the coracoid process and the humeral head on the screen, taking the shortest distance between them. CHD was measured in centimeters, using the calipers on the ultrasound screen (Figs. 1 and 2).

#### ROM-free of pain at shoulder elevation

Range of movement (ROM) free of pain at shoulder elevation was taken using the same procedure as followed for CHD ultrasonography measures, excepting a change in the patient’s position (stand up position). Three measures were taken separated by an interval of 1 minute, and mean was calculated. ROM was expressed in degrees.

#### Shoulder pain and disability index (SPADI)

The Shoulder Pain and Disability Index (SPADI) [19] is a self-administered questionnaire that consists of two dimensions, one for pain and another for functional activities. SPADI total score fluctuates between 0 and 100, with 0 = best and 100 = worst. SPADI has shown to have good internal consistency (overall Cronbach’s alpha = 0.95; for the pain subscale = 0.92; for the disability subscale = 0.93), as well as the ability to detect change over time [20].

### Data analysis

The Statistical Package for the Social Sciences was used for analyzing the collected data (version 23.0 for Mac; SPSS Inc. Chicago, IL). Normality of the variables was visually tested for a Gaussian distribution and additionally tested with a 1-sample Kolmogorov- Smirnov goodness-of-fit test.

For the calculation of reliability of CHI the model or a 2-way mixed consistency intraclass correlation coefficient (ICC) model was used. Hereby a reliability coefficient less than 0.50 was an indication of “poor” reliability; “moderate” between 0.50 and 0.75, “good” between 0.76 and 0.90; and “excellent” over 0.90 [21]. The Standard Error of Measurement (SEM) and the minimal detectable change (MDC) with 95% confidence bounds (MDC95) were calculated. The MDC has been defined as the minimal amount of change that is required to distinguish a true performance change from a change due to variability in performance or measurement error [22]. To the best of knowledge, there is no studies reporting the MDC in the calculation of intra-rater reliability for CHD.

To determine the correlation between CHD at 0 and 60 degrees with SPADI, and ROM free of pain in scapular plane, Pearson correlation coefficient was calculated for normally distributed data, or Spearman’s coefficient in case of absence of normality. Strong correlation was defined as values greater than 0.7; between 0.5 and 0.7 correlation was considered moderate; between 0.3 and 0.5 was considered weak correlation [23].

## Results

A total sample of 87 patients (71% women) was assessed. Sample characteristics are presented in Table 1.

**Table 1:** Sample characteristics

	Mean (SD)
Age (years)	43.9 (9.1)
SPADI (0–100)	59.7 (19.2)
ROM-free o pain (degrees)	93.1 (33.9)
Duration of symptoms	
	3–6 months (26.4%)
	6–12 months (13.8%)
	+12 months (59.8%)

Mean values for CHD at both 0 and 60 degrees are shown in Table 2.

**Table 2:** Intra-rater reliability for CHD at 0 and 60 degrees of shoulder elevation

*n* (87)	mean(SD)	ICC^a^	SEM	MDC_95_
CHD at 0 degrees	1.03 (0.26)	0.988 (0.982–0.992)	0.04	0.11
CHD at 60 degrees	0.94 (0.27)	0.989 (0.984–0.993)	0.04	0.11

Intrarater reliability: *ICC* intraclass correlation coefficient (^a^single measure), *SEM* Standard error of measurement-based on single measure ICC, *MDC95* Minimal Detectable Change with 95%, *CI* based on single measure ICC

### CHD Intra-rater reliability

Intrarater reliability for CHD shown excellent values at both 0 and 60 degrees of shoulder elevation (Table 2).

### Association between CHD with shoulder pain-function and shoulder-ROM free of pain

Correlations between CHD, SPADI and shoulder ROM are shown in Table 3.

**Table 3:** Correlations between coracohumeral space measured by CHD at 0 and 60 degrees of shoulder elevation, and SPADI and shoulder ROM free of pain

	SPADI	SEoE^a^	ROM	SEoE^b^
CHD at 0 degrees	−0.24^*^	18.61	0.23^*^	32.12
CHD at 60 degrees	−0.15	19.16	0.19	32.10

^*^: statistically significant (*p* < .05)

^a^SEoE: Standard error of the estimate (SPADI as dependent variable)

^b^SEoE: Standard error of the estimate (ROM as dependent variable)

A poor correlation was found between CHD and SPADI at both 0 and 60 degrees of shoulder elevation. Furthermore, a poor correlation was found between CHD measurements and active ROM-free of pain at shoulder elevation.

## Discussion

The first aim of this study was to determine the intra-rater reliability for CHD measured by US in patients suffering from anterior chronic shoulder pain. The results showed an excellent reliability for both 0 and 60 degrees of shoulder elevation. The second aim was to analyze the level of association between CHD and shoulder pain-function as well as shoulder ROM free of pain. Poor associations were noted between the outcomes obtained.

To the best of our knowledge, this is the first and largest study reporting CHD measurements, by means of US, in people suffering from chronic anterior shoulder pain. This study provides results in response to the lack of quality studies in the field of coracohumeral reliability, measured by US. Our findings demonstrated excellent intra-rater reliability for CHD at 0 and 60 degrees (0.98), which are in consonance with Tracy et al. [14] who found an ICC of 0.89 at 0 degrees, in a smaller sample of 19 participants free of shoulder pain. Likewise, Oh et al. [13] achieved intrarater reliability greater than 0.90, in patients with rotator cuff tears. However, the position used to measured CHD (cross arm position) in both studies was different in comparison to the present study. The excellent values achieved for CHD measurements are similar to those obtained in similar studies reporting AHD also measured by US, in patients with shoulder pain [24, 25]. These promising findings are supported by different factors that were considered in the present study in order to improve the quality of the results: (1) the ultrasound examiner was blind to the affected shoulder before measurements were taken; (2) a wash out period of one minute between measurements, allowing patients to move freely between these measurements; (3) no landmarks were used on the skin in an attempt to make every measurement independent with respect to the others; 4) the ultrasound examiner was fully qualified. With respect to the normative values for CHD in people with shoulder pain, our results showed values of 1.03 (0.21) cms at 0 degrees of shoulder elevation, and 0.95 (0.25) cms at 60 degrees. Only one study [14] has reported CHD using US, obtaining values of 0.70 (1.4) cms, although CHD was taken in adduction and internal shoulder rotation. This position reduces CHD and so, makes the comparison between findings difficult. MRI has also been used in the assessment of CHD. Specifically, one study has reported values of 0.72 cms [5] in maximal shoulder internal rotation, while with shoulder neutral rotation, values of 1.12 (0.33) cms have been found [10], which are in consonance with the results from this paper. Our values were similar in CHD at 0 degrees of shoulder evaluation (1.03 ± 0.21 cms) to those obtained by Oh et al. [13] (1.01 ± 0.21 cms), but in different patient samples (anterior shoulder pain versus full rotator cuff tear).

To the best of our knowledge, this is the first study investigating the relationship of CHD values, shoulder pain-function and ROM. It is important to establish the possible association between anterior shoulder pain and CHD measured by US, as well as with active shoulder ROM-free of pain, which could indicate treatments in one direction or another. Our results showed an absence of correlation between CHD and both, SPADI and ROM-free of pain. There are possible underlying mechanisms to explain the low association between CHD, pain and function, and active shoulder ROM-free of pain. Anterior shoulder pain is not a homogenous entity. It seems to appear as a combination of intrinsic factors (for example age, tendon histology and genetics), and extrinsic factors, which are those more closely related to CHD, such as anatomic differences for humerus lesser tuberosity and coracoid process [6, 8]. Also, anteversion, internal humeral rotation [7], and a history of chronic overuse of persisted flexion, adduction and internal rotation shoulder position [9]. The controversy in regard to the exact pathomechanics and biomechanical causes of shoulder pain is reasonable. This study only shows the level of association between the CHD and the symptoms referred by the patient, not a cause-effect relationship. Since anterior shoulder pain is multifactorial in character, the CHD could only weakly explain the pain perceived and ROM of the patient. Moreover, the chronic character of shoulder pain suffered by the patients included in the present study, could mean the confluence of other possible explanation factors, such as the presence of peripheral-central sensitization, that has been previously reported in shoulder injuries [26]. The present study can only speculate about the real influence of these factors since they were not measured.

There are some limitations that should be taken into consideration. Firstly, inter-rater reliability for ultrasonography measures was not determined; hence results should be taken with caution. Secondly, the difficulty in classifying shoulder pain disorders could mean that heterogeneity is present in the analyzed sample. Hence, previous studies have remarked on the lack of uniformity and reliability in the current diagnostic classification system for shoulder pain [27, 28]. Thirdly, CHD is a two dimensional measurement of a three dimensional space. Therefore, any compromise of this space cannot be completely quantified by the measurement of CHD in isolation. This should be taken into account. Furthermore, the clinical value of CHD must not be outrightly rejected in the clinical assessment of shoulder pain. Finally, due to convenience sample analyzed in this study, results cannot be generalized to other populations.

This study provides promising results regarding the excellent intra-rater reliability of US in the determination of CHD that quantifies the CHI. Moreover, normative values for CHD at both 0 and 60 degrees of shoulder elevation in patients with chronic shoulder pain have been identified. However, the real role of the CHD in the explanation of pain severity, alteration of shoulder function and limitation of ROM, in patients with anterior shoulder pain, is not sufficiently clear yet. Hence, future studies should be focused on the determination of its real importance along with other intrinsic and extrinsic factors. This could determine whether it should be considered as a prognostic factor for chronic anterior shoulder pain, and whether it could be an essential factor to guide physical treatments. Furthermore, a standard patient position should be agreed upon when using US as this would make possible comparisons between studies possible.

## Conclusions

In patients with chronic anterior shoulder pain, there is poor association between CHD, and shoulder pain and function, as well as with shoulder ROM-free of pain. Hence, clinicians should consider, not only increasing this space, but also other possibilities in their therapies, when patients with anterior shoulder pain are treated. However, the results should not be generalized to other populations.

## Abbreviations

AHD: Acromiohumeral distance
CHD: Coracohumeral distance
CHI: Coracohumeral interval
GP: General practicioner
ICC: Intraclass correlation coefficient
MRI: Magnetic resonance imaging
ROM: Range of movement
SPADI: Shoulder pain and disability index
US: Ultrasonography
